# Practical Departments

**Published:** 1902-06-28

**Authors:** 


### PRACTICAL DEPARTMENTS.

### INVALID CARRIAGES.

Messrs. Reading, who have had 60 years' experience in removing invalids, both by road and rail, were among the exhibitors at the Medical, Surgical, and Hygienic Exhibition recently held at Queen's Hall. At their show rooms we have inspected an invalids' omnibus, with a suspended couch, seats for four persons, lavatory, etc. The trolley on which the couch is placed runs out at the back of the omnibus on rails, and, if necessary, the couch can be carried to the patient's bedroom, so that, however long the journey, there need be no further moving until the destination is reached. We also saw a coach, on the same plan, but lighter and smaller, and with seats for only two persons; the same arrangement for removing the couch is made, through the back of the carriage. There is a door in the side as well, and the vehicle has the appearance of a private brougham. These are for hire, and those built for sale are of a lighter make, though on the same plan.

The provision of a motor ambulance has been suggested to Messrs. Reading; this, they assure us, would be a very costly undertaking, both because the building of a motor is expensive to begin with, and also on account of the higher wages of the driver. From the invalid's point of view, it would be an almost perfect mode of transit, for the noise and jolting would be reduced as low as possible, especially if a high rate of speed could be maintained. In the present stage of motor-history, however, there are certain risks attached to the experiment, and if any part of the machine were to fail serious consequences might result. Messrs. Reading's address is 15 Ridinghouse Street, Langham Place, London.

